# Maize *Aspergillus* section *Flavi* isolate diversity may be distinct from that of soil and subsequently the source of aflatoxin contamination

**DOI:** 10.1007/s12550-024-00532-7

**Published:** 2024-04-22

**Authors:** Bwalya Katati, Stan Kovács, Henry Njapau, Paul W. Kachapulula, Bas J. Zwaan, Anne D. van Diepeningen, Sijmen E. Schoustra

**Affiliations:** 1 Laboratory of Genetics, Wageningen University and Research, Wageningen, The Netherlands; 2 Mycotoxicology Laboratory, National Institute for Scientific and Industrial Research, Lusaka, Zambia; 3 School of Agricultural Sciences, University of Zambia, Lusaka, Zambia; 4 Biointeractions and Plant Health, Wageningen University and Research, Wageningen, The Netherlands

**Keywords:** Aflatoxin, *Aspergillus*, Cyclopiazonic acid, Maize, Soil

## Abstract

*Aspergillus* section *Flavi* (*Flavi*) is a diverse group of fungal species whose common members include *A. flavus* and *A. parasiticus*. These are well-known for the production of aflatoxin (AF) B and G and other toxic metabolites, like cyclopiazonic acid (CPA). They are saprophytic soil dwellers and also become crop opportunistic epiphytes. The consequence is contamination of the crop with mycotoxins, such as carcinogenic AF. We investigated the *Flavi* community structure of maize and that of their surrounding soil, including their mycotoxigenicity. Furthermore, we investigated the link of the maize *Flavi* diversity with preharvest maize AF levels. The study was carried out in four selected districts of Zambia, in a low rainfall zone. The *Flavi* characterisation was triphasic, involving morphological (colony colour and sclerotia formation), metabolic (AF and CPA production) and genetic (calmodulin gene polymorphism) analyses. *Flavi* abundance was determined by dilution plate technique on modified rose Bengal agar. Results showed that *Flavi* communities on maize and in soil differed. Maize had a higher *Flavi* species diversity than soil. *A. parasiticus* dominated the soil community by frequency of field appearance (85%), while maize was dominated by *A. minisclerotigenes* (45%). CPA-producers with or without AF production dominated the maize (65%) while producers of only AF (B/G) dominated the soil (88%). The ratio between maize *A. parasiticus* and *A. minisclerotigenes* abundance seemed to have had a bearing on the levels of AF in maize, with a ratio close to 1:1 having higher levels than a pure community of either *A. parasiticus* or *A. minisclerotigenes*.

**Supplementary Information:**

The online version contains supplementary material available at 10.1007/s12550-024-00532-7.

## Introduction

The genus *Aspergillus* is subdivided into various sections of species. *Aspergillus* section *Flavi* (*Flavi*) is a group of diverse fungal species some of whose members are common contaminants of maize. Common members include species *Aspergillus flavus* and *Aspergillus parasiticus*, which are known for the production of aflatoxins (AFs) B and G, as well as other metabolites (Amaranta et al. 2021; Frisvad et al. 2019) such as aflatrem, cyclopiazonic (CPA) and kojic acids. They are saprophytic soil dwellers that may become epiphytic on crops such as maize, groundnuts or cotton (Diener and Davis 1987). The consequence is contamination of such crops with mycotoxins such as the carcinogenic AFs (IARC 2012).

Soil is the generally agreed reservoir for *Flavi* (Boyd and Cotty 2001; Jaime-Garcia and Cotty 2004, 2010; Kachapulula et al. 2017) serving as the primary inoculum for *Flavi* on crop (Diener and Davis 1987). *Flavi* communities on crops and in soil have been explored before (Donner et al. 2009; Elsie et al. 2016; Garber and Cotty 2014; Sweany et al. 2011; Thathana et al. 2017). Considering that soil is a niche for mycotoxigenic *Flavi* that may subsequently infect crops growing on such soil, *Aspergillus* biocontrol agents (non-aflatoxin producing isolates of *Aspergillus flavus*) are generally applied to the soil, to outcompete mycotoxigenic strains after transfer to the crop. This can therefore be translated as the ability to determine the *Flavi* that could be present on the maize ear on basis of the isolates present (including those artificially applied) in the soil. Previous studies have extrapolated the risk of *Flavi* and AF contamination of crops, based on the *Flavi* community of the (untreated) soil in which those crops would be planted (Donner et al. 2009; Kachapulula et al. 2017; Njoroge et al. 2016; Thathana et al. 2017). In Zambia, for example, it has been demonstrated that the cultivated areas for maize and groundnuts generally harbour *Flavi* species, including toxigenic strains of *A. parasiticus* and *A. flavus*, which pose a risk to maize AF contamination (Kachapulula et al. 2017). Similarly, a study by Donner et al. (2009) indicated that the frequency of AF contamination of maize in Nigeria is likely related to the soil propagules of *Flavi*. However, without artificial perturbation through biocontrol, the soil *Flavi* community may possibly not be an accurate means to predict the risk of maize infection with *Flavi*, despite soil being the generally agreed primary source of inoculum for maize *Flavi* infection. This is considering that soil and maize are two different ecological niches for fungi, which may imply potential variations in type of species dominating soil and maize. For example, species of *Flavi* not detected in the soil due to low abundance, may in fact proliferate on the maize and be highly detected if maize is their ecological niche. This could result in differences in *Flavi* community structures between soil and maize.

With respect to maize in Zambia, the community structure of *Flavi* has been investigated at the time of maize storage (Kachapulula et al. 2017; Kankolongo et al. 2009; Mukanga et al. 2010). *Aspergillus flavus* and *A. parasiticus* were found to be the two most important species contaminating maize and groundnuts*.* The *Aspergillus* species reported in these three studies were *A. flavus*, *A. parasiticus*, *A. niger* and *A. tamarii. Aspergillus flavus* and *A. parasiticus* were the most important members and are both species of concern with regards to the production of AFs. Although aflatoxigenic species contaminating maize in Zambia have been documented, other currently unreported species in the *Flavi* section, which also produce AFs, include *A. aflatoxiformans*, *A. minisclerotigenes*, *A. nomius*, *A. pseudotamarii* and *A. sergii* (Frisvad et al. 2019). In order to better assess the species diversity of *Flavi* community, it would be useful to employ a polyphasic approach of characterisation of isolates, using genetic, morphological and metabolic identification means (triphasic). Although this would also depend on dilution plating and suffer limitations in the case of low abundance species, determining the species with aid of their partial genetic architecture, supported by preliminary morphological screening and metabolic characterisation, would help provide a comparatively higher species resolution and differentiation. For example, *A. oryzae* has been found to be a closer relative of *A. minisclerotigenes* (Kjaerbolling et al. 2020), while previously reported to be a closer relative of *A. flavus* (Chang et al. 2006; Cleveland et al. 2009). Some studies have reported morphotyping misidentification of some *Aspergillus* species whose naming was later corrected upon genetic verification (Balajee et al. 2006; Tam et al. 2014).

Whether or not the previously unreported *Flavi* species in Zambia such as *A. aflatoxiformans* and *A. minisclerotigenes* would be species of concern may depend on their ability to significantly infect maize and thereby contribute to the severity of AF contamination of the crop, especially in the field. For example, dominance of *A. minisclerotigenes* and *A. aflatoxiformans* in an ecosystem, through their detection on maize, would be a subject of concern due to the species’ ability to produce AF B and G variants as well as CPA (Frisvad et al. 2019). CPA has been suggested to be a pathogenicity factor in *Flavi* for maize infection (Chalivendra et al. 2017). This would imply that species producing CPA may likely infect the maize more readily than non-CPA producers and thereby increase the chances of maize AF contamination due to such *Flavi* infection. It should also be noted that CPA is known to have potential for human toxicity (Chang et al. 2009; Goeger et al. 1988; Riley et al. 1992) including an effect on the human monocytes (Hymery et al. 2014). Its negative effects on livestock have been documented (Burdock and Flamm 2000; Byrem et al. 1999), which include increased mortality and reduced weight gain (Domingues et al. 2021; Nishie et al. 1987) as well as the undesired hepatic weight gain (Malekinejad et al. 2010). Furthermore, the negative effects of CPA on broilers have been found to be additive when in combination with AF leading to increased liver and kidney weights in test animals (Domingues et al. 2021; Smith et al. 1992).

With respect to AF contamination of maize, it is known that abiotic factors like temperature and water activity influence the contamination level. However, maize batches from the same germplasm in a given area, under the same weather conditions, can still incur different AF levels. Attributing this simply to heterogeneity in fungal infection of the crop by AF-producing strains could be an oversimplification of the contamination dynamics. Recent explanations for the discrepancy in AF levels within portions has been attributed partly to interaction between toxigenic and atoxigenic *Flavi* isolates. In this case, the atoxigenic *Flavi* could potentially degrade AF produced by the toxigenic counterparts (Maxwell et al. 2021), which would hence, lead to lower AF contamination in some maize batches as compared to others. Furthermore, it should be noted that it has been demonstrated that toxigenic *Flavi* can also degrade AF besides its degradation by the atoxigenic (Katati et al. 2023a). However, it is not known whether or not the *Flavi* species abundances on maize relative to each other could be an attribute to the differential contamination of AF in the maize.

Objectives of this study were (1) to compare the *Flavi* community in soil and on maize. We predict that the *Flavi* community structures between soil and maize are different from each other, with maize having a higher diversity than soil. The prediction is on basis that of the commonly known *Flavi* species with ability to infect maize, a good number are able to produce CPA (*A. aflatoxiformans*, *A. flavus*, *A. minisclerotigenes*, *A. oryzae*, *A. tamarii*, *A. sergii*) compared to those largely unable to produce it (*A. nomius*, *A. parasiticus*, *A. sojae*) (Frisvad et al. 2019). CPA has previously been reported to be a pathogenicity factor for *Flavi* infection of maize (Chalivendra et al. 2017). Another objective was (2) to investigate the influence of *Flavi* diversity on AF levels in preharvest maize. We predict that *Flavi* species on Zambian preharvest maize are more diverse than previously reported, and expect to find additional species capable of producing AF. Furthermore, we hypothesise that the ratio between species capable of producing AF influences the resulting AF levels in maize. The latter hypothesis is on basis that due to the competition for particular niche between two similar species, the mycotoxigenic strains may incline to produce the metabolite AF due to interspecies competition in defence of an ecological niche.

Significance of this study aligns with the desired enhanced food security and safety that AF continues to threaten in Zambia, the subregion and globally. In line with this, several countries in sub-Saharan Africa including Zambia, have been rolling out the biological control of the AF (Bandyopadhyay et al. 2016) by using atoxigenic variants of *Flavi* through the biopesticide AflaSafe (https://www.aflasafe.com; accessed 31st October 2023). The rollout of such a program also requires the deliberate intelligence to be able to predict maize *Flavi* infection propensity by better understanding the community structure between *Flavi* on maize and soil. If the soil and maize community structures mirror each other, then it becomes feasible to predict specific areas that would need consented biocontrol intervention based on soil *Flavi* structure and the risk of translocation of *Flavi* from soil reservoirs. On the contrary, the evidence of niche specialisation of *Flavi* between soil and maize informs a biocontrol programme that focus for isolation of maize AF biocontrol agents (atoxigenic variants of *Flavi*) may best be done from maize and not soil. Further importance of this study is that should a significant number of CPA-producers be found to infect maize, then this informs such a biocontrol programme on *Flavi* species that are more likely to infect maize and pose an AF contamination risk. The CPA-producers of concern in this case would include species such as *A. aflatoxiformans* and *A. minisclerotigenes*, due to their ability to produce B and G variants of AF besides the CPA, a suggested maize pathogenicity factor for maize infection by *Flavi*.

## Materials and methods

### Study area

**Fig. 1:**
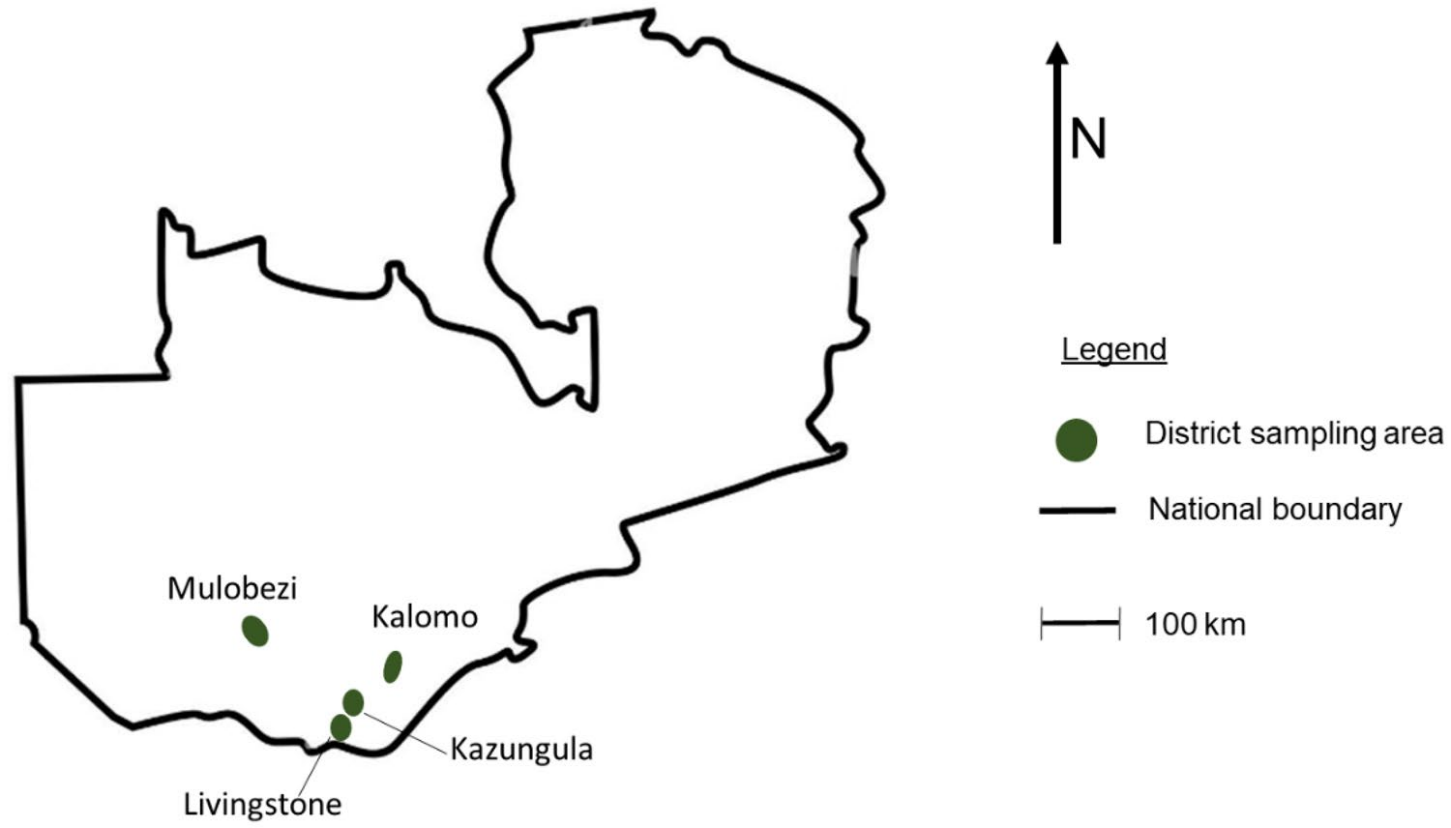
The map of Zambia showing sampling locations of maize and soil under 2018/2019 maize growth season from the southerly districts (Kalomo, Kazungula, Livingstone, Mulobezi)

The study area comprised selected districts of Zambia under agroecological zone 1 (Fig. 1) within latitude 16° 36′ 54′′ S to 17° 46′ 40′′ S and longitude 24° 49′ 57′′ E to 26° 31′ 35′′ E. Soil samples were collected just before maize harvest from the sites, fields, points and time from which maize samples had been collected in our previous investigation which partly involved the study of *Aspergillus* abundance on maize (Katati et al. 2023b). This study’s area of choice (Fig. 1) was selected on basis that *Aspergillus* on maize had largely been detected in this area (*n* = 20 fields) during its 2018/2019 maize cropping season in this previous investigation that employed high-throughput DNA sequencing.

### Determination of *Flavi* community structure

To determine the community structures, both maize and soil *Flavi* were characterised by a triphasic approach. As detailed below, this involved appraisal of phenotypic traits through macro-morphological screening (colony colour and sclerotia), and partial metabolic profiling (aflatoxin, AF, and cyclopiazonic acid, CPA). This was coupled to genetic characterisation based on partial sequencing of the calmodulin (*CAM*) gene with reference isolates used as yardstick to estimate correctness of assignment of species identity. For some verifications, the beta-tubulin gene was used. The *Flavi* abundances and frequencies were determined by dilution plate technique.

**Fig. 2:**
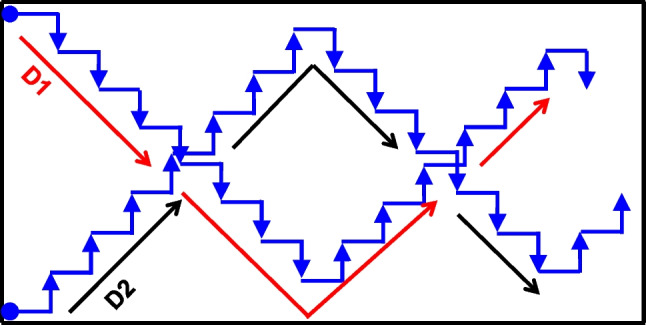
Sampling scheme for soil samples. Black boundary shows an example of a maize field perimeter (not drawn to scale; fields varied in size between 1.0 and 2.5 acres) within which soil (and concurrently maize) samples were collected. ‘D1’ and ‘D2’ with black and red arrows are a schematic representation of the transects from which soil portions were collected to generate a composite sample for the field. Blue arrow heads indicate stop point from which a scoop of soil (along with a maize ear) was collected (not drawn to exact number of steps), avoiding collecting soil/maize from the field edges. The Two blue dots indicate the start points for counting of steps before collecting a soil portion/maize ear. A total of 15 scoops per transect were collected. We presume transect D1 an D2 are same

Collection of the soil is as shown in the scheme in Fig. 2 based on method by Jaime-Garcia and Cotty (2010). The soil subsamples from the two transects per field were combined into one composite soil sample of approximately 1.5 kg. The composite samples were dried (42 ± 2 °C, 48 h), while in cotton bags in a forced-air oven dryer (model D-6450, Heraeus, Hanau, Germany), to prevent any growth of fungi. Soil clumps, if present, were crushed by hitting the bags, then material sieved through mesh (1 mm). The sieved part was then thoroughly homogenised by coning and quartering, scooping from different positions a total of 40–45 g subsample into a sterile 50-ml polypropylene tube.

Soil *Flavi* were isolated on modified rose Bengal agar medium (MRB) as described by (Cotty 1994). A 15 g of homogenised subsample was transferred to a sterile 250 ml bottle, followed by addition of 30 ml of 0.05% sterile Triton-X (0.5 × dilution) to generate an initial extract. The contents were shaken using a sideways shaker (GFL model 3018, Society for Laboratory Technology, Burgwedel, Germany) at 200 rpm for 15 min. Next, 1 ml of soil suspension was transferred, while agitating, to a sterile 10-ml glass test tube containing 4-ml sterile deionised water (0.1 × dilution). A further 1 ml of initial extract was transferred to a 1.5-ml microcentrifuge tube for later downstream enumeration of *Flavi*. The 0.1 × dilution was serially diluted up to 1 × 10^−3^. Next, 150 µl of each suspension was plated on MRB and spread with the help of sterile 3-mm glass beads (20 to 30 beads per plate). The 0.5 × extract was spread using a sterile glass rod as the beads could not readily move in the near paste extract. All MRB incubations were carried out at 31 °C (3 days, dark). For maize samples, the *Flavi* isolates had previously been isolated on MRB for enumeration of *Flavi* on maize in our previous investigation (Katati et al. 2023b). These had similarly been extracted by shaking (40 g kernels/40 ml sterile 0.05% Triton-X solution) prior to serial dilution plating on MRB. For isolate cleaning, 30% (or at least three isolates if fewer than 10) of maize isolates on MRB with characteristics of *Flavi* were randomly selected and sub-cultured on potato dextrose agar (PDA) immediately after their initial isolation on MRB from our previous study. For soil, > 50% isolates were transferred to PDA (*n* = 36 recovered). Isolates were sub-cultured at least twice on the PDA for isolate cleaning by single spore passaging. Spores from purified isolates were stored in replicates in glycerol-agar for downstream determination of toxigenicity of isolates by thin layer chromatography (TLC) with HPLC verification.

#### Characterisation of the *Flavi* isolates

##### Macro-morphological characterisation

**Fig. 3:**
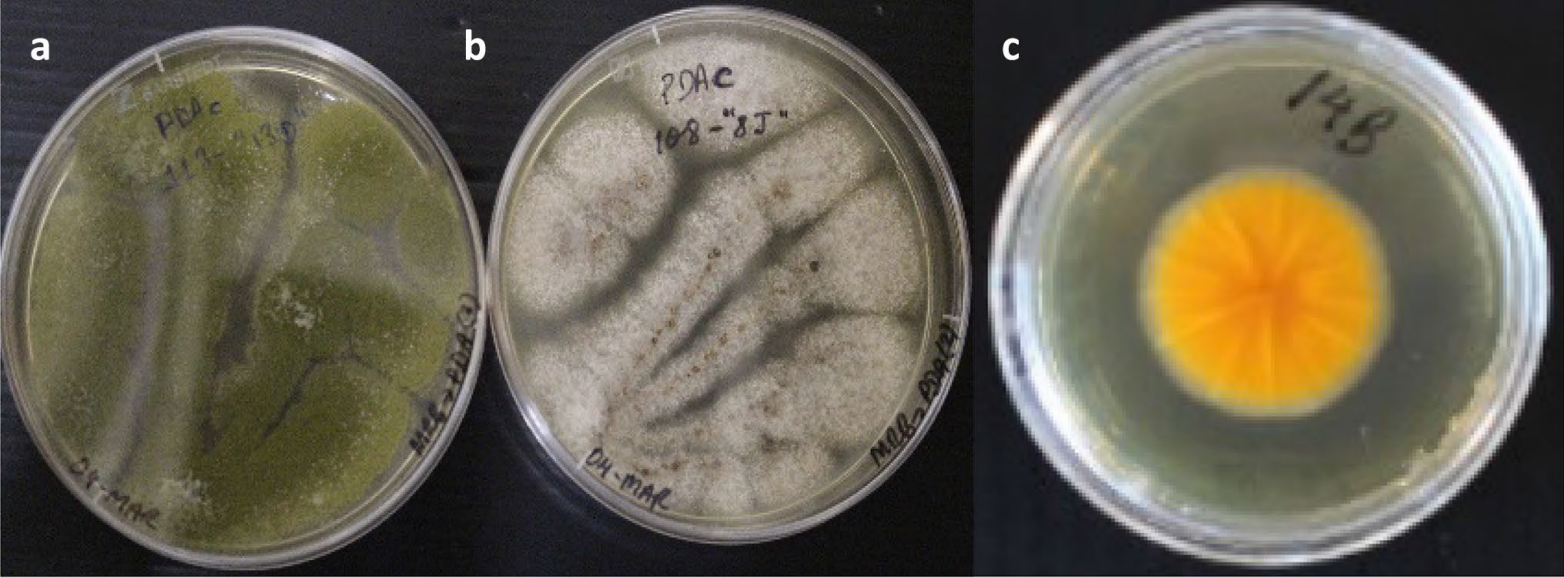
Passaged *Aspergillus* section *Flavi* isolates from MRB onto PDA medium 90-mm petri dishes (**a** and **b**). Macro-morphology shows the end of a 7-day incubation (25 °C, dark) on the PDA. **a** *A*. *parasiticus* isolate MLV13D (short code 13D), with characteristic green shade). **b** *A*. *minisclerotigenes* isolate MKZ08J, with characteristic cream-white colour and visible brown sclerotia. **c** AFPA medium (36-h incubation, 31 °C, dark) on a 35-mm petri dish. Orange reverse characteristic of macro-morphology of all isolated species in maize belonging to *Aspergillus* section *Flavi*. Picture shows *A. parasiticus* isolate MLV14B

Pure wildtype isolates from maize (*n* = 112 recovered) were plated on *Aspergillus flavus*/*parasiticus* agar (AFPA) medium (Pitt et al. 1983) on 35-mm petri dishes for preliminary screening of *Flavi*. Both soil and maize pure isolates (*n* = 148) were plated on potato dextrose agar (PDA) medium in 90-mm petri dishes and incubated at 25 °C (7 days, dark). All isolates were scored for colony colour on PDA medium as well as production or non-production of conspicuous large brown sclerotia on the medium. The maize isolates producing an orange colour on the reverse side of the AFPA plates (Fig. 3 ‘C’) were ready for genetic and metabolic characterisation without further screening, as likely subsets of *Aspergillus* section *Flavi*.

##### Metabolic characterisation

**Fig. 4:**
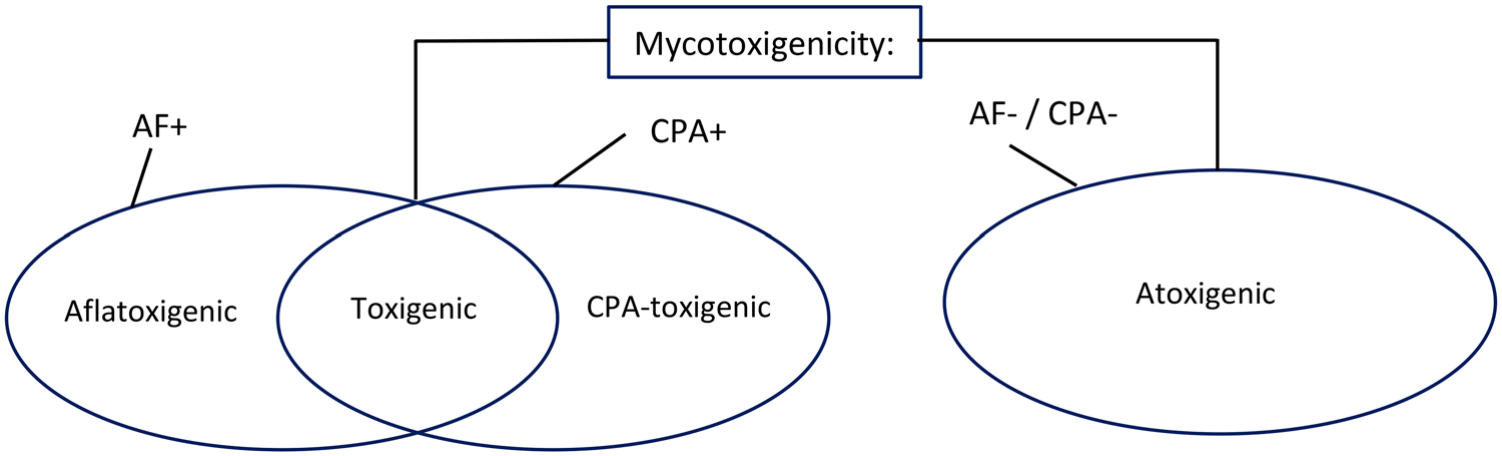
Assignment of mycotoxigenicity to isolates. AF, aflatoxin B and/or G; CPA, cyclopiazonic acid; positive ( +) sign indicates production, while negative sign ( −) indicates non-production of the metabolite

The metabolic profiles were established based on production of AF and CPA by the isolates. Assignment of mycotoxigenicity (production or non-production of AF B/G or CPA) for the isolates is as described in Fig. 4.

**Fig. 5:**
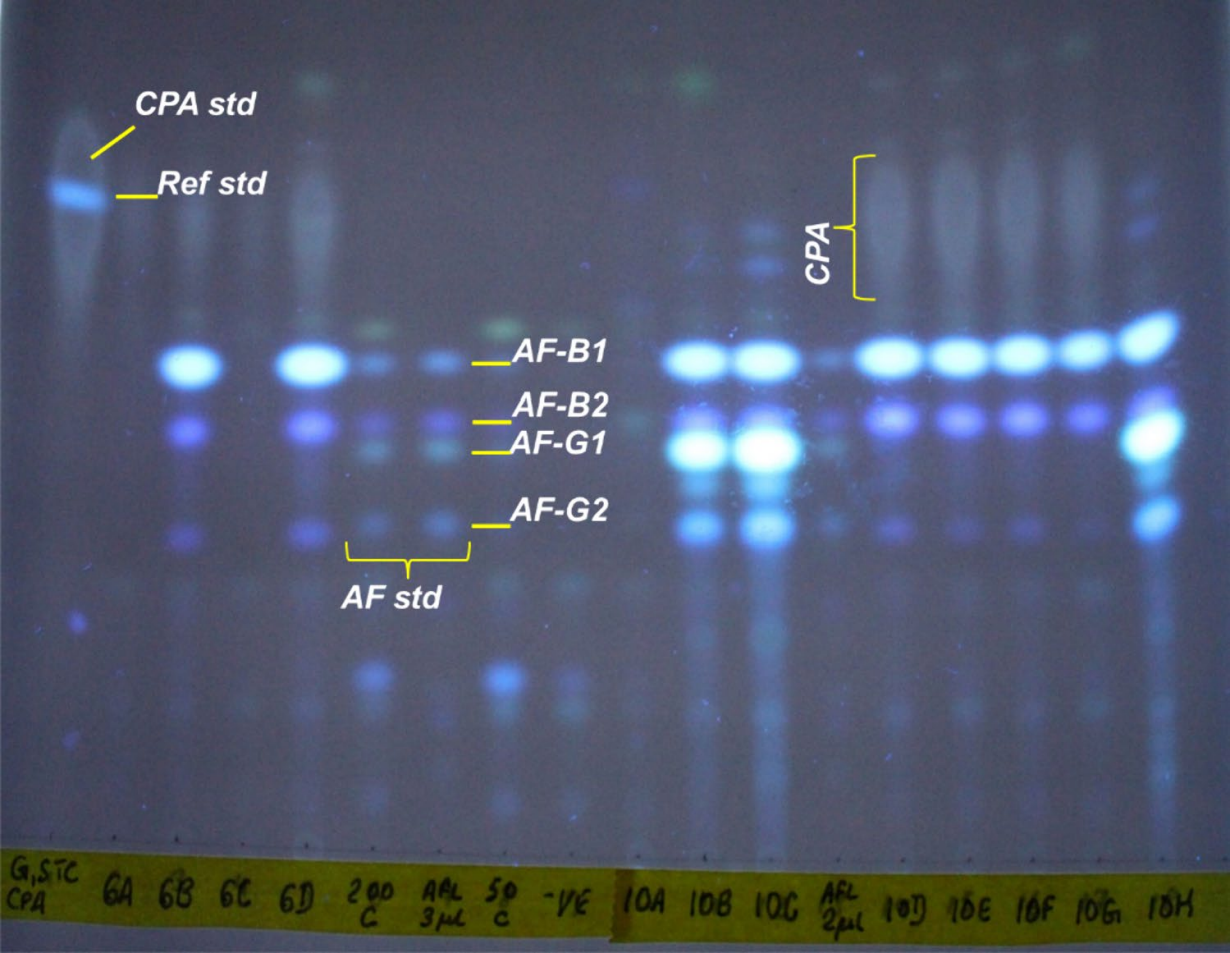
Example of a developed Thin Layer Chromatography plate visualised under UV (312 nm) to qualitatively determine aflatoxin (AF) B_1_, G_1_, B_2_, G_2_ as well as cyclopiazonic acid (CPA) from metabolite extracts of maize *Flavi*. *A. parasiticus* bands of AF-B and -G toxins without CPA are shown in lane ‘10B’ and ‘10C.’ *A. minisclerotigenes* bands are shown in lanes ‘10F’ and ‘10G,’ producing AF-B and -G (faint) and CPA bands. Lane ‘200_C’ is a spiked AF standard in medium that was not inoculated with spores. Lane ‘Afl_3μl’ contains pure AFB_1_, AFG_1_, AFB_2_, AFG_2_ standard, mixed. Lane ‘G,STC_CPA’ is a pure CPA standard spotted together with a griseofulvin standard (griseofulvin is normally used for the determination of the Retardation factor of the metabolites on a TLC plate). Mycotoxigenicity of soil isolates was similarly analysed

For both maize and soil, AF and CPA profiles were generated by thin layer chromatography (Fig. 5) with further verification of the AFs production by HPLC (Agilent Infinity II 1260 Series, Agilent Technologies, Santa Clara, CA, USA). Isolate aflatoxin biosynthesis induction was initially done on yeast extract sucrose (YES) medium (Abdollahi and Buchanan 1981), following the protocol we used in our previous investigation (Katati et al. 2023a). This was prior to verification of toxigenicity on maize grain as medium. This was done in order to establish similarities/differences in metabolic profiles for the triphasic characterisation. For verification of atoxigenicity of isolates that did not produce AF on YES upon HPLC verification, maize grain was used as the AF-inducing medium as described in vitro by Probst and Cotty (2012). Briefly, each pure isolate was inoculated on 45 g sterilised maize grain of 29% moisture content in sterile flasks. Inoculation density per flask was 1000 µl of 1 × 10^6^ spores/ml fungal isolate suspension. Incubation was done at 31 °C (14 days, dark). AF was extracted by wet milling of the maize kernels in 90 ml of 80% MeOH, blending at high speed in a 1L blender (Waring® Commercial, Stamford, CT, USA). Extract was filtered (fluted filter paper vicam® part number 31240) then diluted 1:1 with deionised water. Next, an aliquot of the extract was filtered (0.22 µm, nylon), then 40 µl injected into an HPLC (Agilent Infinity II 1260 Series, Agilent Technologies, Santa Clara, CA, USA). Reversed-phase AF elution was done in isocratic mode under the same HPLC conditions as described in our previous investigation (Katati et al. 2023a). YES AF extraction was done by dichloromethane partitioning coupled with methanol:water (1:1 v/v) reconstitution, following the same protocol from our previous investigation. From the reconstituted material, 2 × 2 µl extract was spotted on a 20 × 20 cm TLC plate (Silica gel 60 without fluorescence, Supelco, MO, USA) alongside AFB_1_, AFB_2_, AFG_1_, AFG_2_ and CPA standards. The TLC plate was developed in 150 ml toluene:ethyl acetate:90% formic acid (5:4:1, v/v) solvent system. The TLC plate was dried for 40 to 60 min under a fume hood and visualised on a UV transilluminator (Model TFX-35 M, Vilber Lourmat, France) at 312 nm (Fig. 5). Frequency of toxigenicity was determined as percentage quantity of isolates producing AF and/or CPA. Isolates were qualitatively assigned metabolic profiles as positive or negative by production of AFB, AFG and CPA. All TLC negative isolates were verified by HPLC using metabolites generated from the YES medium or maize grain.

##### Genetic characterisation

For both maize and soil samples, the stored spore suspension of each isolate was retrieved from storage (− 20 °C) and diluted to 1 × 10^4^ spores/ml with sterile Milli-Q water. The diluted spore suspension replicate per isolate (*n* = 148 maize and soil isolates) was spotted on a sterilised cellophane disc immersed on the surface of MEA medium in a 60-mm petri dish. Samples were incubated for 2 to 3 days (25 °C, dark) allowing just enough fresh mycelia to grow. The fresh dry mycelia were carefully harvested from the disc using a sterile spatula and transferred to a 2-ml sterile microcentrifuge tube (Eppendorf, Hamburg, Germany) containing about five 3-mm sterile glass beads. DNA from the mycelia was extracted using the CTAB breaking buffer (2% hexadecyl trimethyl ammonium bromide, 1.4 M NaCl, 20 mM EDTA, 100 mM Tris HCl, pH = 8.0). Briefly, the tubes of mycelia were first placed in liquid nitrogen (3 to 5 min) then immediately vortexed on a bead beater (FastPrep-24™ 5G, M.P. Biomedicals, LLC, Santa Ana, USA) for 10 s at 30 beats/s. The freezing and beating was repeated then 500 µl CTAB breaking buffer immediately added followed by brief vortex. Next, 2 µl proteinase-K solution (15–20 mg/ml, Qiagen, Hilden, Germany) was added per tube and briefly vortexed. Tubes were incubated at 65 °C for 1 h with mild shaking (300 rpm) on a thermomixer (Comfort, Eppendorf, Hamburg, Germany). Next, 500 µl chloroform:isoamyl alcohol (24:1, v/v) solution was added per tube in a fume hood and mixed gently by inversion ten times. Tubes were centrifuged in a fume hood for 15 min at 25,000 × *g* in a microcentrifuge unit (model 5417R, Eppendorf, Hamburg, Germany) at room temperature. The separated water phase was carefully pipetted off (300 µl) into a new microcentrifuge tube, discarding chloroform phase. Next, 300 µl of ice-cold isopropanol was added per tube, briefly mixing by inversion, five times. The tubes were placed in a freezer at − 20 °C for 20 min (or overnight when a pause in extraction was unavoidable). Tubes were then immediately centrifuged at 25,000 × *g* for 15 min in a temperature-controlled microcentrifuge unit (model 5417R, Eppendorf, Hamburg, Germany) at 4 °C. All liquid was decanted then 300 µl of absolute ethanol added per tube and centrifuged at 25,000 × *g* (room temperature, 5 min). Liquid was decanted then 300 µl 70% ethanol added per tube. Tubes were re-centrifuged at 25,000 × *g* at room temperature for 5 min and the liquid decanted. Tubes were completely dried (overnight, dark) with caps open. The extracted DNA was then reconstituted in 50 µl sterile DNA-/RNA-ase free Milli-Q water. DNA concentrations were measured with a Nanodrop spectrometer (model 2000 ThermoScientific, Wilmington, DE, USA).

For the Sanger sequencing, the purified template DNA concentration of each sample was normalised to 10 ng/µl with sterile Milli-Q water. The DNA was then amplified by PCR for the partial calmodulin gene using primer pairs Cmd5/Cmd6 (forward: 5′-CCG AGT ACA AGG AGG ARG CCT TC-3′, reverse: 5′-CCG ATR GAG GTC ATR AGG TGG-3′) (Hong et al. 2006); and alternative primer set CF1/CF4 (forward: 5′-GCC GAC TCT TTG ACY GAR GAR-3′, reverse: 5′-TTT YTG CAT CAT RAG YTG GAC-3′) (Peterson et al. 2005) used whenever Cmd5/Cmd6 did not amplify. The two primer sets have different binding sites but amplify a homologous target site (PCR product sizes: Cmd5/Cmd6 ~ 580 bp; CL1/CL4 ~ 750 bp). For partial sequencing of the beta-tubulin gene for verification of identity of isolate’s species type were necessary, the following primer set was used in the PCR amplification: T10/Bt2b (forward: 5′-ACG ATA GGT TCA CCT CCA GAC-3′; reverse: 5′-ACC CTC AGT GTA GTG ACC CTT GGC-3′). The 25 µl PCR reaction mixture per tube was composed of 15.9 µl H_2_O (sterile Milli-Q) and the following reagents from Promega™ (Madison, WI, USA): 5 µl buffer (5 × Go green), 1 µl dNTPs, 0.1 µl Taq polymerase (GoTaq) and the above primers from Biolegio™: 1 µl forward primer (10 µM), 1 µl reverse primer (10 µM). Template DNA was 1 µl of the fungal DNA solutions (10 ng/µl). The amplification was done in a thermal cycler (model T100™, Biorad Laboratories Inc., Hercules, CA, USA) under the following conditions: × 1 step of hot start at 95 °C for 3:00 min; × 31 steps of DNA denaturing at 95 °C for 0:45 min; × 31 steps of primer annealing at 57 °C (primer set Cmd5/Cmd6) or 47 °C (primer set CL1/CL4) or 55 °C (primer set T10/Bt2b) for 0:30 min; × 31 steps of strand elongation at 72 °C; × 1 final step of elongation at 72 °C for 4:00 min. Gel electrophoresis was performed on 1% agarose gel amended with ethidium bromide to aid DNA visualisation. The electrophoresis was performed in a gel submarine at 70 V, then DNA bands visualised on a Biorad gel imaging system (Biorad Laboratories Inc., CA, USA) at 312 nm.

All PCR products were purified using the Qiagen PCR product purification kit (Qiagen, Hilden, Germany) as prescribed in the protocol. Unlike PCR products amplified with the Cmd5/Cmd6 primer set, multiple bands instead of the desired one band were produced by the PCR products generated with the CF1/CF4 primers set. Therefore, for purification of the PCR product of CF1/CF4, the target band was excised from the agarose gel before the product clean-up. Hence, for the clean-up: a clean scalpel was used to excise about 40 to 80 mg of the target band from the agarose gel. Excision was done with the aid of UV visualisation of the ethidium bromide-stained band, limiting the total exposure time of the DNA to UV to 5 s. Next, 2 µl NT1 buffer was added per a 1-mg excised agarose in a sterile 1.5-ml microcentrifuge tube. Tubes were incubated for 10 min at 50 °C, vortexing every 3 min. The dissolved mixture was transferred to a NucleoSpin® Gel and PCR Clean-up column attached to a 2-ml collection tube. Contents were centrifuged at 11,000 × *g* for 30 s and the same protocol of the Qiagen PCR product purification kit followed as for non-excised DNA.

Following the purification, library preparation and Sanger sequencing was performed at Eurofins Genomics (85560 Ebersberg, Germany, https://eurofinsgenomics.eu/), using the same primers used for the PCR amplification.

For the bioinformatic analysis, the raw FASTA files from the sequencing data were inspected and cleaned in MEGA7 version 7.0.26 (Kumar et al. 2016). Front and back ends of the sequence outputs of low quality, based on inconsistent peak width or height, peak overlap, inconsistent distances between nucleotide letters, were denoised by their exclusion. The evolutionary analyses were also conducted in MEGA7, and the evolutionary history inferred using the maximum likelihood method based on the Tamura-Nei model (Tamura and Nei 1993). All positions with less than 95% site coverage were eliminated, which means fewer than 5% alignment gaps, missing data, and ambiguous bases were allowed at any position. Assignment of taxa to the aligned sample FASTA files was achieved using the GenBank database (https://www.ncbi.nlm.nih.gov) with the help of reference isolates (Supplemental Table S1).

#### Frequency and abundance of *Flavi* in soil and maize and community structure

*Flavi* abundance was determined as colony quantities (CFU/g soil or maize). This was determined by dilution plate technique on modified rose Bengal agar (MRB) (Cotty 1994). The frequency of each species was determined as proportion, in percentage, of number of times a species appeared across the sampled fields to total number of fields (*n* = 20).

For determination of the community structure, a phylogenetic tree was constructed in MEGA 7 from the saved sequence alignments described above. For generation of bootstrap support, 500 replicates were used. The constructed tree (maximum likelihood) with branches was further processed as an ‘nwk’ file in software R (R Core Team 2023) version 4.3.2 using package ggtree (Yu 2020). The metabolic profiles were linked to the phylogenetic tree in ggtree using the packages ComplexHeatmap (Gu et al. 2016) and gplots (Warnes et al. 2024). The community diversity of *Flavi* in soil and maize was determined using the Shannon index, deduced from eight species detected, namely *A. flavus*, *A. oryzae*, *A. minisclerotigenes* and *A. parasiticus*, which were the most detected members; then *A. sergii*, *A. transmontanensis*, *A. sojae* and *A. krugeri* as the less detected (*n* = 1 field). Outside section *Flavi*, only one isolate was detected as *A. tubingensis* belonging to section *Nigri*.

### Influence of *Flavi* diversity on preharvest aflatoxin contamination

#### Proportion (%) of species capable of producing aflatoxin

From the characterised *Aspergillus* species, we determined % of isolates capable of producing aflatoxin (AF) in maize across the sampled locations. This was determined as the percentage of afla-/toxigenic species against the overall *Aspergillus* species on maize using geometric mean. It was presumed that AF in maize will only originate from the species capable of producing AF, present on the maize. Determining the proportion of isolates capable of producing AF may, however, be only used to assess the risk of the AF contamination as not all AF producers detected may possibly be responsible for the detected AF contamination on the maize. Therefore, the link between diversity and observed levels of maize AF was investigated further as described immediately below.

#### Influence of ratio of species capable of producing aflatoxin on maize aflatoxin levels

The species ratio of the two most abundant afla-/toxigenic species (able to produce AFB and/or AFG with or without CPA) on maize was determined. Afla-/toxigenicity was based on TLC profiles as described above. Abundance was based on CFU counts, with species identification aided by Sanger sequencing. We selected samples from fields that produced at least six confirmed *Flavi* CFUs per MRB petri dish (*n* = 10). The number of isolates per petri dish were counted without dereplication of clonal isolates. The isolates were then categorised as afla-/toxigenic or non-aflatoxigenic based on the TLC AFB/AFG profiles. To fit a model to explain the influence of the species ratio on AF levels, we generated the independent variable ‘X’ as sum of the proportion ratio of the two most afla-/toxigenic species on the plate. ‘X’ was determined by adapting the Shannon diversity index to our scenario as below and defined as a modified Shannon index (*H*_*m*_): 

1 $$\text{X}=\sum \limits^{2}_{\text{i}=1}[(\text{n}_{\text{i}}/\text{N})^{*}\text{Ln(n}_{\text{i}}/\text{N})]$$

where, *n* = number of *A. parasiticus* and *A. minisclerotigenes* as the most frequent afla-/toxigenic isolates of *Flavi* on maize across fields, overall; *N* = number of the total *Aspergillus* species in a specific field determined by triphasic characterisation.

The dependent variable Y is the total aflatoxin level in the field maize on a logarithmic scale. For this purpose, we obtained the aflatoxin levels in maize from our previous study (Katati et al. 2023b) which had been determined by HPLC.

### Data analysis

Using a triphasic approach, we selected for each field any isolate that had at least one difference in either the morphological (colony colour and presence/absence of conspicuous [large] brown sclerotia), metabolic (AF-B/G and CPA) or genetic lineage (calmodulin, *CAM*, gene sequence) from other isolate(s). Isolates with no difference in any of the three characteristics were scored as clonal, and hence dereplicated by selecting only one isolate per clone(s) for each field. Using clonal isolates, for this approach, would not yield any information on the phylogenetic tree to help differentiate the community structure between maize and soil.

The characterisation was followed by the quantification of soil and maize species abundance (CFU/g). In addition, a phylogenetic tree was built to describe the *Aspergillus* species distributions in soil and maize. Maize *Aspergillus* species mycotoxigenicity was determined as the proportion of species in a district not producing or producing particular metabolite(s) AF and CPA in relation to the total number of *Aspergillus* species in the location. For this purpose, geometric mean coupled with geometric standard error was used to determine the quantities, due to the heterogenous distribution of the CFU data. CFU counts to determine *Aspergillus* species abundance were also used to determine the influence of the *Aspergillus* species ratio on field AF contamination between *A. minisclerotigenes* and *A. parasiticus* (equation 1). For the determination of the influence of species abundance ratios (*X*) on maize AF levels (*Y*), a linear model of *Y* = *f*(*X*) was fitted using Microsoft® Excel® version 365. Significance of the model (*P*-value) and the value of its regression line (*R*^2^) was then determined. For statistical comparisons, CFU/g data were log-transformed, with prior conversion of zero values to calculated limits of detection, LOD, (equation 2) based on (Magnusson 2014). The LODs were 0.57 CFU/g soil, and 1.90 CFU/g maize based on the limit of blank from the lowest detected CFU/g values and the sample blanks (*n* = 7 replicates per ecological niche).

2 $${\text{LOD}}= \frac{3*\mathrm{ Stdev}}{\sqrt{n}}$$

## Results

### Determination of *Flavi* community structure between maize and soil

A total of 148 isolates (maize *n* = 112, soil *n* = 36) were characterised using a triphasic approach. From the dereplicated isolates, 58 were selected (maize, *n* = 33; soil, *n* = 25) leaving only unique isolates within a field, for purpose of assessment of species differential distribution between soil and maize. The characterisation, as described below, was on basis of colony (colour) and sclerotia morphology for the morphological characteristics; AFB, AFG and CPA profiles for metabolic characterisation, then DNA sequence clustering based on the partial calmodulin (*CAM*) gene polymorphism for the genetic characterisation. Estimation of correctness of inference of species identity was aided with reference isolates (Supplemental Table S1; Supplemental Fig. S3). The identified isolates were mainly *A. parasiticus*, *A. minisclerotigenes*, *A. flavus* and *A. oryzae*. Other *Flavi* isolates with very low abundances as well as frequency of detection across sampled fields were *A. krugeri, A. sergii*, *A. sojae* and *A. transmontanensis*. Only one isolate outside section *Flavi* was detected (*A. tubingensis*—section *Nigri*).

#### Characterisation of *Flavi* in soil and on maize

##### Morphological characterisation

**Fig. 6:**
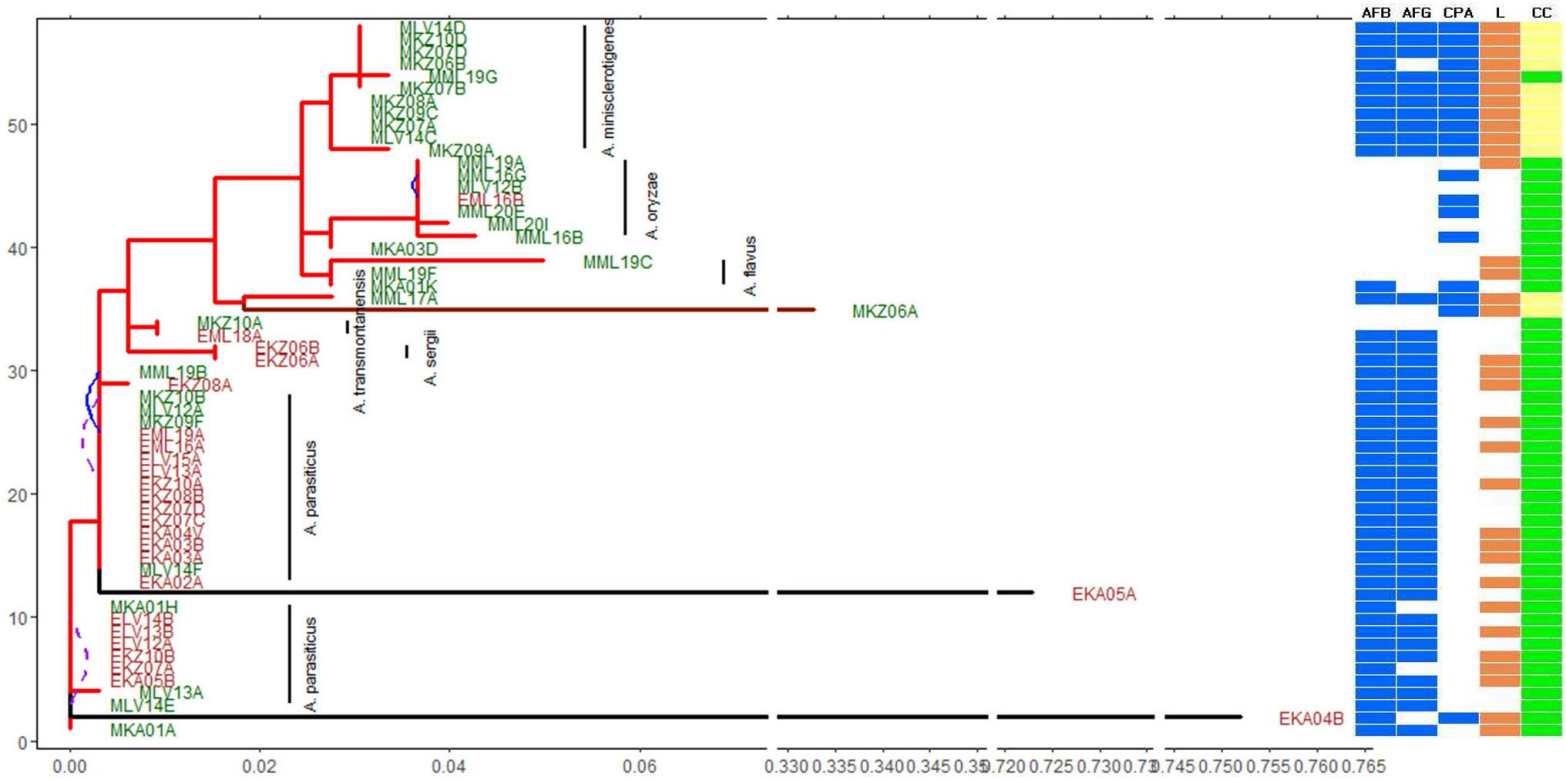
Phylogeny of *Aspergillus* species isolates detected in soil (brown with starting letter ‘E,’ *n* = 25) and maize (green with starting letter ‘M,’ *n* = 33) of selected southerly districts of Zambia in the 2018/2019 maize growth season. Blue solid lines (*n* = 2) show corresponding soil and maize isolates from the same field (same numerical value) having no phenotypic (metabolic/morphological) differences and no sequence divergence on the partial calmodulin gene. Purple dotted lines (*n* = 2) show corresponding soil and maize isolates from the same field having no sequence divergence on the partial calmodulin gene but with phenotypic difference recorded. Rest of isolates had some form of within field differences between the soil and maize *Aspergillus* species. The metabolic characteristics of each species based on AFB_1_ and/or AFB_2_, AFG_1_ and/or AFG_2_ and CPA are qualitatively indicated as blue (produced by the species) or white (not produced by the species). Morphological characteristics on the tree are indicated as L, with brown signifying conspicuous brown sclerotia characteristic of L-morphotype, and CC for colony colour with light yellow signifying cream white morphology or green shade signifying green morphology. Unclustered species on the tree include MKA03D—*A. flavus* and MML17A—*A. krugeri*. Black for tree branch indicate a longer branch on *x* -axis than light red. Isolates EKA04B, EKA05A and MKZ06A are highly divergent with branch lengths truncated. The soil isolates (including clones), being dominated by *A. parasiticus*, mainly produced the AFB and AFG, without detectable CPA (Fig. 6). Only two (non-clonal) CPA producers were detected in soil (Fig. 7). The proportion of CPA producers on maize was significantly higher than in soil (*P* = 0.020)

Morphologies of the isolates are shown in the phylogenetic tree (Fig. 6). Overall, *A. minisclerotigenes* produced a cream-white shade, except for MML19G, which in addition had a middle green shade. All *A. minisclerotigenes* isolates produced the conspicuous brown sclerotia. All *A. parasiticus* produced a green shade. Production of brown sclerotia was not a distinctive trait for *A. parasiticus*, as half of the (non-clonal) isolates investigated produced the brown sclerotia while the rest did not. All *A. flavus* and *A*. *Oryzae* isolates produced a green shade on their mycelium due to formed green conidiospores. There was no distinction in production of visible brown sclerotia in these isolates. All isolates’ colony diameters on PDA (7 days, 25 °C, dark) were ≥ 25 cm (single inoculation at centre of petri dish).

##### Genetic characterisation

On maize, a total of 33 *Aspergillus* species isolates (non-clonal) were determined. Isolates were identified as *A. minisclerotigenes* × 11, *A. parasiticus* × 9, *A. oryzae* × 7, and *A. flavus* × 3. The other two isolates detected were one each for *A. transmontanensis* and *A. Krugeri*. One isolate outside section *Flavi* was detected as *A. tubingensis* (section *Nigri*) in one field. *A*. *flavus* and *A. oryzae* had close genetic lineage. In this study, we found *A. minisclerotigenes* to be a closer relative to *A. flavus*, followed by *A. oryzae* (Fig. 6). In soil, 25 *Flavi* isolates (non-clonal) were determined. From these, 19 were *A. parasiticus*, two *A. Sergii* and one each as *A. oryzae*, *A. flavus*, a highly divergent *A. sojae*, and *A. transmontanensis*.

##### Metabolic characterisation

*A. parasiticus* characteristically produced aflatoxin (AF) B and G, and did not produce CPA (Fig. 6) as would be expected. On the other hand, *A. minisclerotigenes* was a producer of AFB, AFG and CPA, and was exclusively detected on maize. On maize, CPA producers were also found to be aflatoxigenic in 3/4 locations (districts). Isolates in the district Mulobezi largely had no detectable AF produced.

#### Soil and maize *Flavi* relative abundance and community structure

The overall spread of the *Flavi* species between the two ecological niches, soil and maize, was as follows: *A. parasiticus* was the most frequently detected species in soil, surpassing all other species encountered in soil combined (Fig. 8). In contrast, *A. minisclerotigenes* was exclusively detected on maize, with none detected in soil (Fig. 6). Overall, there was a higher species variation in maize compared to soil, as shown in Fig. 8. This is supported by a higher Shannon diversity index for maize (1.34) compared to soil (0.80). This is despite soil having a higher abundance of *A. parasiticus* than maize. Some species were detected in very low abundance in either soil or maize. These were *A. krugeri*, *A. sergii*, *A. sojae* and *A. transmontanensis*. Only one isolate outside section *Flavi* was detected (*A. tubingensis*—section *Nigri*).

**Fig. 7:**
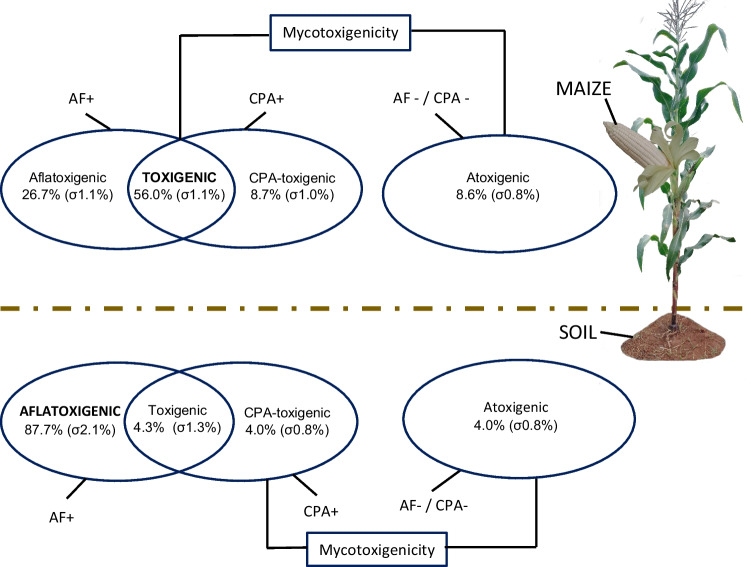
Proportion of mycotoxigenicity of *Flavi* detected in soil and on maize kernel. The proportions are expressed as percentages and are based on the geometric mean and geometric standard error (*σ*). Bold in caps shows the larger percentage of the mycotoxigenicity in the given niche

While *A. parasiticus* was predominantly detected in soil, CPA producers, with or without AF, including *A. minisclerotigenes*, were predominant on maize (Fig. 6), with only two CPA-producing isolates (EKA04B, EML16B) detected in soil in this region (Fig. 6). *A. parasiticus*, however, was abundantly detected in both the soil and on maize grain. *A. oryzae* and *A. flavus* were all detected on maize, except for one isolate of *A. oryzae* (EML16B) and one *A. flavus* (EKA04B) in soil. The non-producer isolates, like the CPA producers, were only reported on maize and none in soil. The four predominant species on maize in order of decreasing frequency of field detection were: *A. minisclerotigenes* (4/4 districts; 9/20 fields), *A. parasiticus* (4/4 districts; 7/20 fields), *A. oryzae* (3/4 districts; 6/20 fields) and *A. flavus* (3/4 districts; 4/20 fields). In this sampling season and area, *A. minisclerotigenes* and *A. parasiticus* were the most common species found on maize (Figs. 6 and 7), with *A. parasiticus* also being the most common species found in soil.

All *A. parasiticus* isolates for maize and soil produced AF-B and -G without CPA (Fig. 6). Only four non-*parasiticus* isolates (EML18A, EKA05A, EKZ06A and EKZ06B) had such a profile of producing AF-B/G without detectable CPA. The production of all three metabolites, AF-B, -G and CPA, was exclusive to *A. minisclerotigenes*, with the exception of one isolate of *A. krugeri* (MML17A), which also produced the three metabolites. The production of CPA only within section *Flavi*, was exclusive to *A. oryzae*. Atoxigenic isolates (producing none of the three metabolites) were mainly *A. oryzae* and *A. flavus*. There was a significantly higher population of CPA-toxigenic isolates in maize compared to soil, (*P* = 0.020).

### Influence of *Flavi* diversity on preharvest aflatoxin contamination

#### Frequency of aflatoxin-production-capable species

We detected in high frequency *A. minisclerotigenes*, a species previously unreported on Zambian maize, with aflatoxin (AF) producing potential. The overall frequency of species mycotoxigenicity is presented in Fig. 7. If we were to consider districts as a variable, most isolates on the maize were producers of AF compared to its non-production, except for one location (Mulobezi district) with a longitude to the west of the other three locations (Fig. 1), which had a lower frequency of AF producing isolates (Mulobezi *n* = 11/34), compared to the other locations (Kalomo *n* = 16/18; Kazungula *n* = 33/36; Livingstone *n* = 23/24).

#### Influence of aflatoxin-production-capable species ratio on maize aflatoxin levels

When considering the influence of species diversity (ratio between *A*. *parasiticus* and *A*. *minisclerotigenes*) on the levels of AFs produced in maize, a linear regression model fits the data significantly (Fig. 9, *P* = 0.001). The lowest AF content in *Flavi*-contaminated maize was recorded at a ‘modified’ Shannon diversity index (*H*_*m*_) of ‘0’ (only one species between *A. parasiticus* and A. *minisclerotigenes* present, fields 08 and 13). On the other hand, the highest content of AF was recorded when Hm had the highest value (0.72, Fig. 9), also signifying a closer value to a 1:1 ratio between *A*. *parasiticus* and *A*. *minisclerotigenes* relative to the total *Flavi* on a maize sample (fields 10 and 14).

## Discussion

Overall, there were differences in *Flavi* species community structures between maize and its corresponding soil. On maize, the previously unreported *Aspergillus minisclerotigenes* in Zambia had a bearing on the aflatoxin (AF) levels in the preharvest maize in the 2018/2019 maize cropping season.

### Diversity of Aspergillus section *Flavi* (soil and maize)

#### Characterisation of *Flavi* isolates

**Fig. 8:**
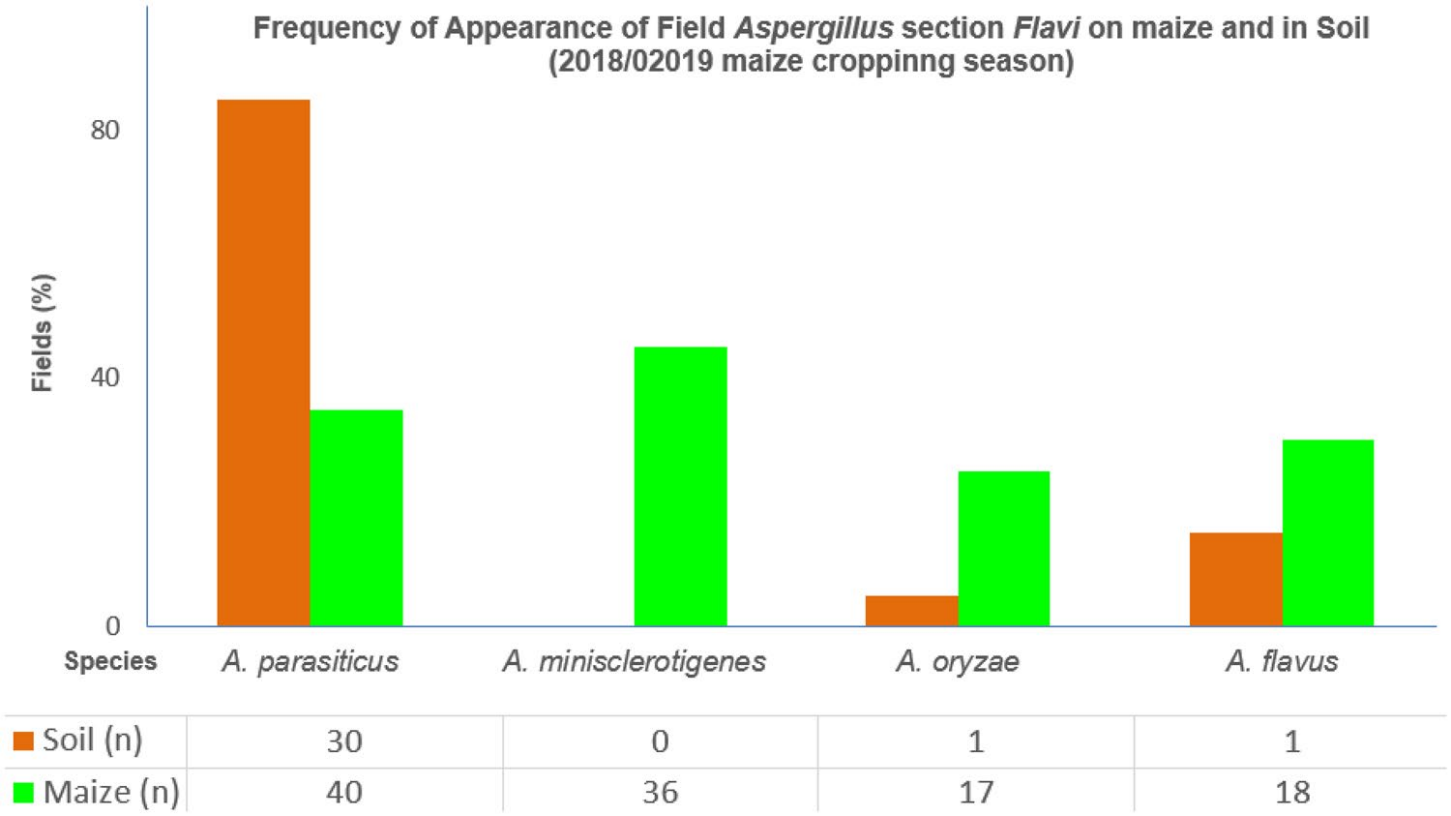
Most frequently detected *Aspergillus* section *Flavi* species on maize and in soil. Frequency is expressed as a percentage of the number of fields in which a particular species is detected over total number of fields (*n* = 20) sampled. ‘n’ is based on total number of the representative proportion of unknown *Aspergillus* species isolates that were passaged for characterisation

Despite a cross-sectional study, the characterisation of the *Flavi* aided by molecular means detected additional important *Flavi* species on Zambian preharvest maize and soil across the four sampled districts than previously reported. The major additions included *A. oryzae* and *A. minisclerotigenes*, which we detected in relatively high frequencies on maize (Fig. 8).

**Fig. 9:**
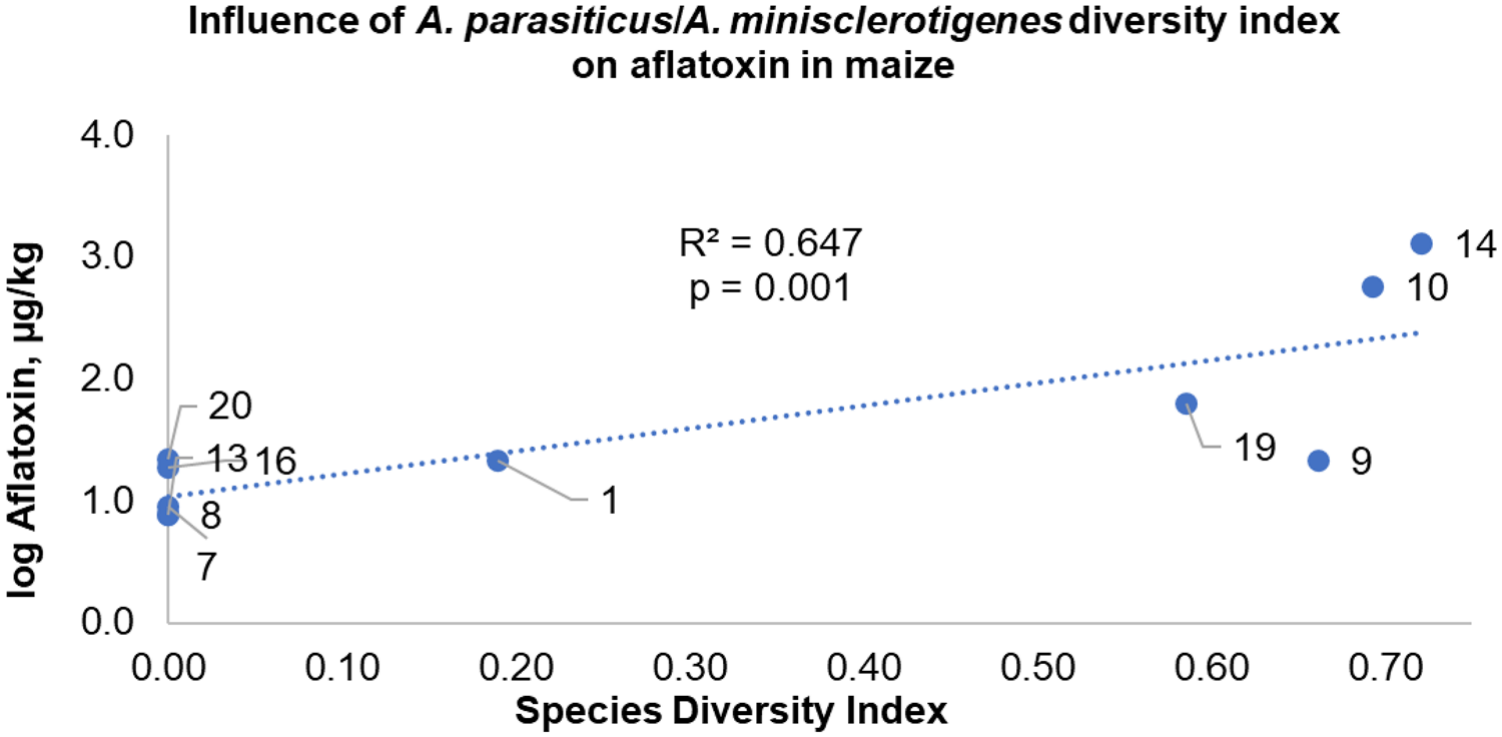
Influence of species diversity on levels of AF in field maize. The digits making the regression line are field numbers (*n* = 10). A modified Shannon diversity index (Hm) equation 1 (Material and methods) was used to calculate species diversity (in an ideal situation, excluding other species, the highest possible *H*_*m*_ value would be 0.69, when the *A. parasiticus* to *A. minisclerotigenes* ratio is 1:1)

Morphologically, *A. minisclerotigenes* consistently produced conspicuous brown sclerotia on PDA, with cream-white vegetative mycelium, sparse conidial heads. Some studies have, however, also shown that *A. minisclerotigenes* can morphologically be misidentified as *A. flavus* (Oloo et al. 2019; Karimizadeh Esfahani et al. 2019). This is observed with our *A. minisclerotigenes* isolate M19G (Fig. 6), which resembled *A. flavus* due to the green shade. Although *A. flavus* isolates mainly produced brown sclerotia in current study, it should be noted that this does not imply *A. flavus* is always a producer of sclerotia. As community distributions of *Flavi* are bound to change with season (Ortega-Beltran and Cotty 2018), isolates of *A. flavus* that do not produce brown sclerotia could, for example, be found to be predominant on preharvest maize or be less dominant in another season. In addition, media could influence the morphology such as production of sclerotia. However, from the production of distinct brown sclerotia on PDA by *A. minisclerotigenes* under the specified conditions in current study, we may infer that the morphology is predominant of this species as described in a previous study of *Flavi* (Frisvad et al. 2019). It is furthermore worth noting that *A. oryzae* can easily be morphologically misidentified as *A. flavus*, or indeed reported as an atoxigenic variant of its close relative *A. flavus* (Chang et al. 2006) (Fig. 9).

We demonstrate that *A. flavus* was a close relative of *A. oryzae*, based on sequence divergence (Fig. 6) in part of the calmodulin gene which resonates with earlier findings by Chang et al. (2006) and Cleveland et al. (2009). Our findings further show that *A. minisclerotigenes* was further away from *A. oryzae* and *A. flavus*. In addition, *A. minisclerotigenes* was further away from *A. oryzae* than it was from *A. flavus* based on the partial *CAM* gene sequence. This is contrary to findings by Kjaerbolling et al. (2020) who demonstrated closeness of *A*. *minisclerotigenes* with *A. orzyae* than *A. flavus*. We attribute these differences to the choice of loci used in the genetic differentiation. However, consensus is that in section *Flavi*, *A. parasiticus* would cluster away from *A. flavus*, *A. orzyae* and *A. minisclerotigenes*.

Metabolically, *A. minisclerotigenes* has been implicated in the notorious levels of AFs detected in Kenyan maize east of the country (Oloo et al. 2019). Identified on Zambian maize in this sampling season, this may sound alarm on the potential danger for high maize AF contamination in Zambia. However, we cannot directly implicate *A. minisclerotigenes*, in the observed field contamination of maize with AF from the sampled locations (Fig. 1) as it may not have been the single species producing the AFs. Further importance of *A. minisclerotigenes* in phytosanitary is that the species is also a categorical producer of CPA (Fig. 6) (Frisvad et al. 2019; Pildain et al. 2008). Beyond plant pathology, an *A. minisclerotigenes* isolate was recently implicated as human keratitis agent in Iran (Karimizadeh Esfahani et al. 2019).

The proliferation of *A. minisclerotigenes* in the current study maize cropping season (2018/2019) may be attributed to a dry spell during the maize growth season which the sampled locations experienced. It may be suggested that such severely dry and hot conditions may have benefited *A. minisclerotigenes* colonisation of maize, just like hotter temperatures seem conducive for its close relative *A. flavus*, which performs better than *A. parasiticus* under those conditions (Ching'anda et al. 2021). This is consolidated by the fact that *A. minisclerotigenes* was not detected under the normal low rainfall conditions for the region from the exact same fields in the 2020/2021 maize growing season (Supplemental Fig. S2), a season that generally had a lower frequency of detected *Flavi* as well as the genus *Aspergillus (*Katati et al. 2023b*).* This also suggests that the observed preharvest AF contamination pattern could have been seasonal, with the potential to recur. It is also plausible that *A. minisclerotigenes* may be present on stored maize, but is unreported due to absence of molecular surveillance. This study demonstrates that the diversity of *Flavi* in Zambian maize is beyond the commonly reported *A. flavus* and *A. parasiticus*. This warrants the need for further seasonal surveillance of preharvest maize *Flavi* by triphasic characterisation of the *Flavi* community structure in order to estimate the diversity of the *Flavi* and subsequently the maize AF contamination risk. The observed high frequency of *A. minisclerotigenes* coupled with its production of both B and G variants of AF and CPA shows that this is a fungus deserving attention in the maize *Flavi* infection scenario.

Although earlier country studies showed that *A. flavus* was the major contaminant of stored maize (Kachapulula et al. 2017; Kankolongo et al. 2009; Mukanga et al. 2010), the likely carry-over of the other detected species in the current study from harvest to storage would imply that more species beyond *A. flavus* can contaminate the crop during storage. We attribute the higher number of species on maize detected in this study compared to earlier country studies to close genetic relationships and morphological similarities of *A. flavus* to species such as *A. oryzae*. This shows the usefulness in coupling phenotypic (morphological/metabolic) screening of *Aspergillus* species with their genetic characterisation. Our overall findings are in agreement with previous country findings indicating that *A. parasiticus* is (one of) the major contaminant of maize (Kachapulula et al. 2017). From other studies of *Aspergilli* on maize, we do note that the previously reported *Aspergillus* species *A. parasiticus*, *A. flavus*, *A. tamarii* and *A. niger* are canonically non-producers of CPA, except for some strains of *A. flavus*. In contrast, the detected maize isolates in the current study, except for *A. parasiticus*, were all capable producers of CPA (all strains of *A. minisclerotigenes* and some strains of *A. oryzae* and *A. flavus*). From our findings, the significant frequency of detection of CPA-producing *Flavi* on preharvest maize may suggest a potential risk of maize contamination with CPA other than AFs, particularly when conditions become favourable. Furthermore, two non-aflatoxin producing isolates of *A. flavus* were found to produce detectable CPA. Such isolates producing CPA are not uncommon. It therefore remains imperative when selecting candidates as non-aflatoxigenic biocontrol strains for AF, that such candidates are screened for non-production of CPA.

From both the morphological and metabolic characterisation, we do see under the conditions of this study that the production of metabolites had a better delineation of the species than the production of (large) sclerotia (Fig. 6).

#### Comparison of soil and maize *Flavi* community structures

Soil is the generally agreed reservoir for *Flavi*. In line with this, for example, past studies have suggested AF contamination events in maize to be likely driven by the toxigenic isolates from soil (Donner et al. 2009; Kachapulula et al. 2017; Njoroge et al. 2016). However, it should not be inferred, for example, that observed high frequency of detection of *A. parasiticus* in soil would mirror the frequency of this species on maize. We demonstrate in our studied districts during the 2018/2019 maize cropping season that the *Aspergillus* species community structure on maize greatly differed from that in soil, with maize having a higher frequency of appearance, per field, of particular *Flavi* species compared to soil (Fig. 8). Furthermore, while all species that had been detected in soil were also detected on maize, some species on maize were in fact not detected in soil. In addition, a soil isolate with no sequence divergence between itself and the corresponding maize isolate was found in only 2/20 fields (Fig. 6), out of a total of 148 isolates screened with 58 per-field-unique strains detected across the 20 fields. This implies that the use of the single calmodulin gene locus met the objective on *Flavi* community study, as differences and not similarities are what were mainly detected. The sufficiency of the single locus used is evidenced by the fact that we detected only the two maize-soil isolate pairs with no *CAM* gene sequence divergence and phenotypic (metabolic/morphological) differences within a field. The difference in community structure between maize and soil in current study resonates with previous study (Sweany et al. 2011). The previous study reported an overall difference between soil and maize isolates, reporting only six out of 16 vegetative compatibility groups of soil *Aspergillus* species to also be present on maize. Therefore, although the risk of AF contamination in maize has at times been estimated based on the *Flavi* community in the soil, we demonstrate that this should be done with precaution as the two *Flavi* community structures can differ. However, with crops like sugarcane, soil could possibly be a predictor for the crop’s *Flavi* as described in the study by Garber and Cotty (2014) that showed an influence of soil *Flavi* community on that of the sugarcane. From the current study, it should be noted that the non-detection of *A. minisclerotigenes* in soil as well as the absence of strains similarity between maize and soil does not immediately undermine grounds that soil is main primary inoculum for *Flavi*. It is plausible in our findings that some species not detected in soil, such as *A. minisclerotigenes* as a possible result of low abundance, may proliferate on maize if that happens to be their conducive niche. This could explain, the dominance of the soil-absent *A. minisclerotigenes* on maize, compared to dominance of *A. parasiticus* in all soils (Fig. 6) of the same fields. *A. parasiticus*, for example, is a poorer coloniser of maize compared to *A. flavus* (Dorner et al. 1999; Horn 2003; Zummo and Scott 1990) and generally produces lower spore densities in air compared to *A. flavus*. As a result, this augments the likelihood that high proportions of *A. parasiticus* in soil may not mirror the proportions on maize. With respect to this, we indeed observe in the current study the high frequency of detection of *A. parasiticus* in soil compared to maize.

Although *A. parasiticus* was the most commonly found soil *Flavi* in our study, it should also be noted that the *Flavi* soil community structure can be latitude-dependent, with higher temperatures likely to favour *A. flavus* over *A. parasiticus* (Ching'anda et al. 2021). For example, studies in Kenya showed that the density of *A. flavus* in soil was higher than that of *A. parasiticus* (Benard et al. 2013; Elsie et al. 2016). This is the opposite with the Zambian scenario (Kachapulula et al. 2017)(Fig. 8), which has a different climate and is at higher latitude than Kenya. The study by Jaime-Garcia and Cotty (2010) indicated that soil in which maize was planted has a tendency to be colonised by *A. flavus*. This again is not the case with the Zambian scenario. It should also be noted that the *Flavi* propagule density may change with time, particularly if collection of soil is done after harvest. At such a time, it is likely that *A. flavus* would dominate the community due to plant residues left in the soil (Boyd and Cotty 2001; Zummo and Scott 1990), which would subsequently have a larger influence on the *Flavi* propagule type and density than weather (Jaime-Garcia and Cotty 2010). Therefore, to better understand *Flavi* niche specialisation (between soil and maize), soil collection may best have to be done at such a time when plant residues have not been ploughed back into the soil such as before harvest, as in the current study, or some months after sowing.

Our findings also show that CPA producers may have a propensity towards maize colonisation as their niche compared to soil dwelling, considering that CPA producers were more frequently detected on the maize compared to soil (Fig. 6). These findings corroborate previous study on maize, which suggested that maize isolates produced more CPA than soil isolates and that CPA producers had a higher propensity to colonise maize kernels (Chalivendra et al. 2017). Therefore, CPA may be necessary for infection of maize by *Flavi* but not AF. The AF may instead be more necessary for the survival in the soil niche as suggested by Sweany et al. (2011) and as observed by a high AF-producing ability of soil *Flavi* (88%; Fig. 7) in the current study compared to maize *Flavi* (17%). In line with findings of the current study, *A. minisclerotigenes*, detected in high frequency, may be a species of concern, being better armed with the metabolic accessories to both colonise the maize ear easier through the CPA, and contaminate the crop in field or post-harvest with genotoxic AFB and AFG as well as the neurotoxic CPA.

#### Influence of *Flavi* diversity on aflatoxin contamination

The high frequency of afla-/toxigenic *Flavi* found on maize (Fig. 7) demonstrates the risk for AF contamination of maize when conditions become favourable. Furthermore, *A. minisclerotigenes*, a species previously unreported on Zambian maize, was detected in high frequency, and all its isolates were producers of genotoxic AFB and AFG and neurotoxic CPA (Fig. 6). This warrants it to be a species of interest and concern in the Zambian food safety landscape on basis that on top of producing AF-B and AF-G, it could also be a more robust coloniser of maize than non-CPA producers. Although the species contributed to the AF contamination risk of the maize based on its field frequency (Fig. 8) and afla-/toxigenicity, this was not sufficient to conclude causal relation between *A. minisclerotigenes* and the AF contamination of the field maize. This is considering that some fields that had *A. minisclerotigenes* did not have detectable AF levels, despite being under the same dry weather pattern as some AF-heavily contaminated fields, a scenario likely driven by the dry spell. As an alternative explanation to the differences in AF contamination due to species present, we report on a simple model correlating the influence of the abundance ratio between *A. parasiticus* and *A. minisclerotigenes* on preharvest maize AF contamination. There was low (< 10 μg/kg) maize AF contamination observed when the ratio between *A. parasiticus* and *A. minisclerotigenes* was close to 0 (actual ≤ 0.2), even under high CFU count of a species. On the other hand, higher AF levels (≥ 20 ug/kg) were recorded in fields with higher ratios described by the modified species diversity index in equation 1. In postulation, this may suggest that as the two most afla-/toxigenic species became more equal in relative abundance to each other, a trigger of higher overall AF production occurred. This could also imply the potential for AF to be a signature of competition between similar species for colonising the kernel. This being the first investigation of such field *Flavi* ratio’s influence on AF, it warrants further studies with a larger sample size to consolidate the findings. Overall, findings show that within the diversity of *Flavi* in preharvest Zambian maize, *A. minisclerotigenes* can be an important contributor to the AF contamination dynamics of the maize.

In conclusion, the difference in the *Flavi* community structure between soil and maize in the current study’s season may demonstrate that the different *Flavi* species may indeed preferentially be adapted differently to the ecological niches, soil and maize, with CPA-producers having a propensity for maize colonisation rather than soil dwelling. On this basis, the risk of infection of maize with *Flavi* and subsequently AF should not be anchored on the community structure of the soil, but rather on the maize community structure. We attribute the observed higher *Flavi* species diversity found on the maize compared to soil to the higher number of species that produced detectable CPA compared to the non-CPA producer *A. parasiticus*, which dominated the soil in this study. Employing a triphasic characterisation, we demonstrate that the maize *Flavi* landscape on Zambian preharvest maize is quite diverse, with high frequencies of the previously unreported *A. oryzae* and the aflatoxigenic and CPA-toxigenic *A. minisclerotigenes*. Lastly, we postulate that the AF levels in the preharvest maize during the study period may have been driven by the ratio of the two most abundant species *A. minisclerotigenes* and *A. parasiticus* capable of producing aflatoxin. We recommend further studies and a larger sample size on this last aspect.

### Supplementary Information

Below is the link to the electronic supplementary material.

Supplementary file1 (DOCX 990 KB)

## Data Availability

Additional data including sequences for aligned *Aspergillus* species and code for the evolutionary analyses is found at https://github.com/bkatati/adiversity. Further data is available from Corresponding Author upon reasonable request.
