# Supplementary material for: Maize Aspergillus section Flavi isolate diversity may be distinct from that of soil and subsequently the source of aflatoxin contamination
Source: Mycotoxin Res. 2024 Apr 22;40(3):351–67. doi: 10.1007/s12550-024-00532-7 (PMC11258066; doi:10.1007/s12550-024-00532-7)
Supplement: Supplementary file 1 — Supplementary file1 (DOCX 990 KB) [file 12550_2024_532_MOESM1_ESM.docx]

- 1. **Supplementary information**

**Supplemental Table S1**. Reference *Aspergillus* section *Flavi* isolates from GenBank (FASTA).

| **Species** | **GenBank accession number *** |
| --- | --- |
| *A. flavus* | CBS501.65 |
|  | MG517996.1 |
|  | MG517989. |
|  | MG518125.1 |
| *A. minisclerotigenes* | MG518009.1 |
|  | MG518021.1 |
|  | MG518022.1 |
|  | MG518083.1 |
| *A. aflatoxiformans* | MG518076.1 |
|  | MG518075.1 |
|  | MG518079.1 |
|  | MG518090.1 |
| *A. oryzae* | EF661506.1 |
|  | EF661507.1 |
| *A. parasiticus* | MG518030.1 |
|  | MG518019.1 |
|  | MG518097.1 |
|  | MG518126.1 |
| *A. sergii* | MG518059.1 |
| *A. sojae* | EF202041.1 |
|  | EF661517.1 |
|  | MG518028.1 |
| *A. transmontanensis* | HM803020.1 |
|  | HM803021.1 |
|  | HM803023.1 |
|  | HM803028.1 |

The following *A. flavus* isolates were added to the analysis as live laboratory reference cultures: “01MS7,” “125GF7,” “125FG8” and “126MS6” obtained from Laboratory. **Reference isolates selected from* ([Frisvad](#_ENREF_21)[et al. 2019](#_ENREF_21)^a^).

**Supplemental figure S2**. Relative abundance of Aspergillus section *Flavi* (*Flavi*) detected on maize from four selected districts (Kalomo, Kazungula, Livingstone and Mulobezi) in the 2020/2021 maize growth season. Relative abundance is expressed as percentage, based on the CFU of particular species to total number of CFUs of detected *Flavi* from the sampled fields (*n* = 20).


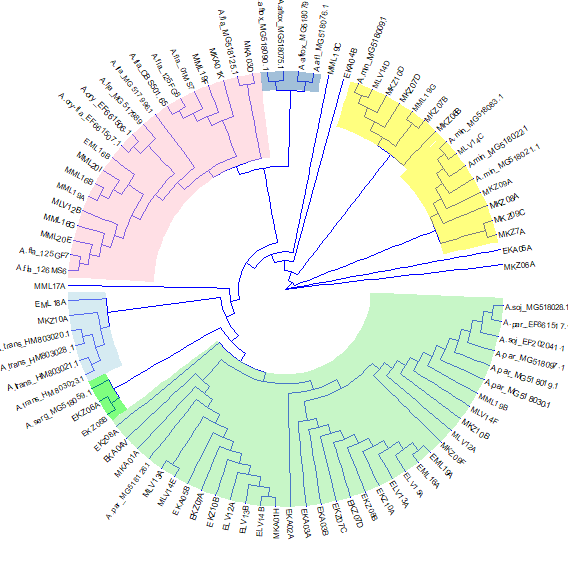
**Supplemental Figure S3**. Phylogenetic tree of estimation of correctness of assignment of isolate species identity based on **GenBank reference isolates** (**Long codes > 6 characters, with prefix ‘A’)**.

Abbreviations: *A.aftox = Aspergillus aflatoxiformans; A.fla = Aspergillus flavus; A.min = Aspergillus minisclerotigenes; A. Ser = Aspergillus sergii; A.soj = Aspergillus sojae; A.trans = Aspergillus transmontanensis**.* Note: The unclustered MKZ06A is *Aspergillus tubingensis* belonging to *Aspergillus section Nigri* and was the only isolate detected outside *section Flavi.*

a Frisvad JC, Hubka V, Ezekiel CN, Hong SB, Novakova A, Chen AJ, Arzanlou M, Larsen TO, Sklenar F, Mahakarnchanakul W, Samson RA, Houbraken J (2019) Taxonomy of *Aspergillus* section *Flavi* and their production of aflatoxins, ochratoxins and other mycotoxins. Stud Mycol 93:1-63. <https://doi.org/10.1016/j.simyco.2018.06.001>
